# Conditional Reprogramming for Patient-Derived Cancer Models and Next-Generation Living Biobanks

**DOI:** 10.3390/cells8111327

**Published:** 2019-10-27

**Authors:** Nancy Palechor-Ceron, Ewa Krawczyk, Aleksandra Dakic, Vera Simic, Hang Yuan, Jan Blancato, Weisheng Wang, Fleesie Hubbard, Yun-Ling Zheng, Hancai Dan, Scott Strome, Kevin Cullen, Bruce Davidson, John F. Deeken, Sujata Choudhury, Peter H. Ahn, Seema Agarwal, Xuexun Zhou, Richard Schlegel, Priscilla A. Furth, Chong-Xian Pan, Xuefeng Liu

**Affiliations:** 1 Department of Pathology, Center for Cell Reprogramming, Georgetown University Medical Center, Washington, DC 20057, USA; nancy.palecho-ceron@georgetown.edu (N.P.-C.); Ewa.Krawczyk@georgetown.edu (E.K.); Aleksandra.Dakic@georgetown.edu (A.D.); Vera.simic@georgetown.edu (V.S.); Hang.yuan@georgetown.edu (H.Y.); Sujata.Choudhury@georgetown.edu (S.C.); Seema.Agarwal@georgetown.edu (S.A.); richard.schlegel@georgetown.edu (R.S.); 2 Department of Oncology, Lombardi Comprehensive Cancer Center, Georgetown University Medical Center, Washington, DC 20057, USA; Jan.Blancato@georgetown.edu (J.B.); ww63@georgetown.edu (W.W.); Yun-Ling.zheng@georgetown.edu (Y.-L.Z.); paf3@georgetown.edu (P.A.F.); 3 Department of Otorhinolaryngology-Head and Neck Surgery, University of Maryland, Baltimore, MD 21201, USA; Fleesie.Hubbard@som.umaryland.edu (F.H.); HDan@som.umaryland.edu (H.D.); Scott.Strome@som.umaryland.edu (S.S.); kcullen@umm.edu (K.C.); 4 Department of Otorhinolaryngology-Head and Neck Surgery, Georgetown University Medical Center, Washington, DC 20057, USA; Bruce.Davidson@georgetown.edu; 5 Inova Translational Medicine Institute, Inova Health System, Fairfax, VA 22031, USA; john.deeken@inova.org; 6 Department of Radiation Medicine, Georgetown University Medical Center, Washington, DC 20057, USA; Peter.H.Ahn@gunet.georgetown.edu; 7 iCryobiol and iFuture Technologies, Shanghai 200127, China; zhou@avantech.cn; 8 University of California at Davis, Sacramento, CA 95817, USA; cxpan@ucdavis.edu

**Keywords:** conditionally reprogrammed cells, patient-derived cancer models, organoids, living biobanks

## Abstract

Traditional cancer models including cell lines and animal models have limited applications in both basic and clinical cancer research. Genomics-based precision oncology only help 2–20% patients with solid cancer. Functional diagnostics and patient-derived cancer models are needed for precision cancer biology. In this review, we will summarize applications of conditional cell reprogramming (CR) in cancer research and next generation living biobanks (NGLB). Together with organoids, CR has been cited in two NCI (National Cancer Institute, USA) programs (PDMR: patient-derived cancer model repository; HCMI: human cancer model initiatives. HCMI will be distributed through ATCC). Briefly, the CR method is a simple co-culture technology with a Rho kinase inhibitor, Y-27632, in combination with fibroblast feeder cells, which allows us to rapidly expand both normal and malignant epithelial cells from diverse anatomic sites and mammalian species and does not require transfection with exogenous viral or cellular genes. Establishment of CR cells from both normal and tumor tissue is highly efficient. The robust nature of the technique is exemplified by the ability to produce 2 × 10^6^ cells in five days from a core biopsy of tumor tissue. Normal CR cell cultures retain a normal karyotype and differentiation potential and CR cells derived from tumors retain their tumorigenic phenotype. CR also allows us to enrich cancer cells from urine (for bladder cancer), blood (for prostate cancer), and pleural effusion (for non-small cell lung carcinoma). The ability to produce inexhaustible cell populations using CR technology from small biopsies and cryopreserved specimens has the potential to transform biobanking repositories (NGLB: next-generation living biobank) and current pathology practice by enabling genetic, biochemical, metabolomic, proteomic, and biological assays, including chemosensitivity testing as a functional diagnostics tool for precision cancer medicine. We discussed analyses of patient-derived matched normal and tumor models using a case with tongue squamous cell carcinoma as an example. Last, we summarized applications in cancer research, disease modeling, drug discovery, and regenerative medicine of CR-based NGLB.

## 1. Traditional Cancer Cell Lines and Animal Cancer Models

Traditionally, cancer research and drug development utilize cancer cell lines, their derived cancer models and genetically engineered mouse models (GEMM). Cancer cell lines have been cultured in vitro for a long time, leading to the acquisition of additional genetic aberrations and epigenetic modifications that can dramatically differ from the original cancers. It has been shown that, even after a few generations, there was a great irreversible genetic divergence between primary tumors and their derived cell lines [1]. Hence, it is not surprising that prediction models for drug response based on the genetic information of cell lines, such as the Genomics of Drug Sensitivity in Cancer [2] and the Cancer Cell Line Encyclopedia [3], frequently fail to predict drug efficacy in the clinic [4]. The success rate of achieving this has been only 1–10% depending on the tissue type and model type [5]. These cell lines have been useful for in vitro experiments to study cancer biology, biochemistry, and drug targets. However, drawing conclusions about how in vitro observations may relate to clinical biology is challenging because cancer cell lines: 1) lack the cellular complexity and architecture of human tumors, which introduces possibility that genetic drift may have occurred after the cell line was established; 2) are not associated with clinical information from the patient; 3) genomic relatedness to the parent tumor is unknown, and molecular characterization including assessment of genomes and transcriptomes of these cell lines, until recently, were mostly unavailable; 4) from diverse racial and ethnic groups and rare cancers are seldom represented in currently available cell lines. Since is extremely difficult to generate and maintain normal tissue-derived cell lines, one option is to use exogenous immortalization methods to keep cells in culture. Interference with the RB/p16 pathway coupled with the induction of telomerase is sufficient for immortalizing many types of primary cells. For example, it is possible to bypass the cell senescence block using viral oncogenes such as SV40 large T antigen or the HPV E6/E7 proteins of the oncogenic human papillomaviruses. However, the resultant cell lines will obviously have aberrant p53 and Rb regulatory pathways [6,7,8,9,10,11]. Alternatively, it is also possible to immortalize primary human adult cells with exogenous cellular genes (hTERT, c-Myc, Bmi-1, cdk4, etc.), but this method leads to disrupted cell differentiation as assayed by mammosphere formation in Matrigel™ [12]. Thus, there is utmost need to identify a new method that can generate cancer cell lines from early stage tumors and from under-represented tissue types and to be able to readily establish corresponding normal tissue-derived cell lines. Being able to have paired normal/cancer cell lines from each patient will improve our understanding of the critical changes in cell biology level as well as enabling the identification of novel drug targets and personalized therapeutic strategies.

Each patient’s tumor is not only heterogeneous but also unique with respect to genetic makeup and signaling. Currently the diagnosis and treatment decisions are based on the histopathological features, morphology and in some instances a restricted panel of immunohistochemical markers that are able to group patients in subgroups. This approach fails to recognize the variations within each subgroup, leading to failure of therapy regimens and recurrence of tumor. In terms of GEMMs, cancers usually develop within a few months to 1–2 years. Even though GEMMs manifest some aspects of oncogenesis of human cancer, cancers developed in GEMM are relatively genetically homogenous. In contrast, many human cancers develop after years of exposure to carcinogens from smoking and environment, and harbor multiple and diversified genetic alterations. This at least partially explains that only approximately 5% of oncology drugs developed based on these models and entering clinical trials are eventually approved by the Food and Drug Administration [13].

## 2. Genomics-Based Targeting Therapies

Similarly, traditionally, cancer treatment takes the anatomy/histopathology- and stage-based one-formula-fits-all approach in which cancer patients with similar histopathology and stages are treated with the same regimen. For example, all stage IV non-small cell lung cancer (NSCLC) patients, including adenocarcinoma and squamous cell carcinoma, are treated with platinum-based doublet chemotherapy with similar and disappointing response rates of less than 30% [14]. Recent developments in and convergence of cancer biology, “-omics” technologies, computational biology and drug development are revolutionizing targeted therapy and leading to a new level of precision cancer medicine in which cancer treatment is designed to target the underlying genetic and epigenetic alterations of individual cancers. With recent developments, the seemingly “uniform” lung adenocarcinoma can be categorized into several subgroups and treated accordingly based on the underlying genetic alterations. For example, response rates of approximately 70% were observed when targeted therapies are used against epidermal growth factor receptor (EGFR) mutations or anaplastic lymphoma kinase (ALK) gene fusion in those patients [15,16,17,18], compared to less than 30% for cytotoxic chemotherapy [14]. However, at this nascent stage of precision cancer medicine, “precision medicine” is still designed based on the studies conducted in cell lines and other cancers, and therefore, the outcomes are usually disappointing in most patients. For example, two recent clinical trials showed that matching molecularly targeted therapies against the underlying patient-specific genetic aberrations was associated with disappointing response rates of 12% (patients with advanced cancer) and 9% (only patients with metastatic breast cancer), respectively [19,20]. Targeted therapy against a known molecular drive in other cancers may not be effective in different cancers. For example, the phosphatidylinositol 3-Kinase (PI3K) inhibitor idelalisib has already been approved for the treatment of relapsed chronic lymphocytic leukemia and follicular B-cell non-Hodgkin lymphoma. Little efficacy was observed when a PI3K inhibitor is used in treating lung squamous cell carcinoma carrying PI3K activation mutations [21]. Currently, the percentage of US patients with cancer who benefit from genome-driven oncology is less than 10%, and functional analyses using patient-derived models are urgently needed for precision oncology [22,23,24].

## 3. Patient-Derived Cancer Models and CRC are Needed for Precision Oncology

In recognition of the limitations of above cancer models, several patient-derived models of cancer (PDMCs), such as patient-derived xenografts (PDXs), conditionally reprogrammed cell cultures (CRCs), organoids, spheroids, induced pluripotent stem cells (iPSC) and others have been recently developed. As in any other model system, each of these platforms has their own advantages and disadvantages in terms of their utilities and their representation of tumor architecture, microenvironment, cellular composition and heterogeneity, stem-differentiation states, growth patterns and responses to treatments, all these merits and drawbacks usually are highly depending upon the initial patient specimen. These approaches are also under active evaluation for future potential in understanding of basic cancer biology and utilities in translational cancer research, especially for drug screening, target identification, and biomarker discovery.

### 3.1. iPS (Induce Pluripotent Stem) Cells

Both embryonic and adult stem cells can be propagated in vitro and these cells retain a normal karyotype and can differentiate into several germ layers, providing great potential for applications in regenerative medicine [25,26]. Several recent review articles summarized applications if iPS in cancer modeling [27,28,29,30,31].

### 3.2. Organoid Cultures

The laboratories of Clevers, Kuo and others successfully established organoid cultures initially from mouse tissues [32,33,34,35,36,37]. These studies have now been extended to human specimens, both normal and tumor. Organoid cultures provide a platform to investigate basic biology for early stage cancers, to identify drug targets, and to study drug resistance [36,38,39,40,41,42,43,44,45]. Unlike monolayer cell lines that are grown on plastic, organoid cultures are established using three-dimensional growth of epithelial cells in Matrigel™. These cultures are fairly stable genetically, can be grown for a long period of time and are not clonal in selection, but rather capture partial heterogeneity of the original tumor. Paired normal and cancer organoids can be established from the same patient samples, thereby providing an opportunity for its application to personalized medicine and regenerative medicine. Precise tumor tissue sampling is very crucial for organoid cultures as this culture system propagates both normal and cancer cells. Additionally, these cultures are more suitable for low-throughput rather than high throughput drug screening [44,45]. Overall it takes 4-6 weeks to provide enough cells for drug screening [46].

### 3.3. Patient-Derived Xenografts (PDX)

In recent years patient-derived xenografts have emerged as a viable system for modeling human tumors in vitro [47,48]. Unlike cell lines, such as lack of stromal components, clonal selection, and genetic drift due to long-term culturing conditions, PDX models cross-talk between stromal components and epithelial tumor cells, appear to have high genetic stability, especially during early passages, and can preserve the molecular and cellular heterogeneity of the primary tumor. In addition, PDX models appear to predict metastatic potential and response too [49]. Currently, PDXs are still widely recognized as a more physiologically relevant preclinical model to standard cell line xenografts. PDX models faithfully recapitulate the original patient genetic profile, gene expression patterns and tissue histology. Despite their benefits, PDX models are limited by their inherent variability, lower throughput and lack of growth in vitro. The ability to generate cell lines from PDX models would enable high throughput chemosensitivity screens, ex vivo genetic manipulation and the development of novel orthotopic models. Development of stable PDX cell lines remains a challenge due to murine stromal outgrowth, lineage commitment and limited differentiation potential. Overall, it takes 2–5 month for PDX expansion at a relatively high cost of mice and their care.

### 3.4. Conditionally Reprogrammed Cells (CRCs)

It remains a challenge to establish a single model system that is rapid, simple to perform, and has a high rate of success. The CRC method we developed at Georgetown meets these needs. The culture method can rapidly convert normal and tumor cells to a state of “reprogrammed stem-like” in which the cells are highly proliferative and maintain their original karyotypes; moreover, the removal of these conditions restores the capacity for cell differentiation [12,50,51]. Thus, we named this method “conditional reprogramming”. As we have described below in the detailed protocols. Briefly, the tissue samples are first evaluated histologically by a pathologist to determine the composition of the sample (i.e., what percentage of the cells are tumor cells). The samples are then dispersed into single cells by enzymatic digestion and plated in a medium containing irradiated Swiss-3T3-J2 mouse fibroblasts (feeder cells) and 10 µM Y-27632. Epithelial colonies are readily observed at two days and usually proliferate rapidly to reach confluence in approximately five days. The conditional reprogramming method is applicable to many epithelial tissues including skin, prostate, lung, breast, kidney, salivary gland and liver cell types and it has recently been extended to neuroendocrine and endocrine tissues. Interestingly, the method seems generally applicable to many mammalian species such as mouse, rat, dog, ferret, horse, and cow [52,53,54,55,56,57]. An important feature is that these cultures can be used for establishing xenografts [12,50] and patient-derived xenograft cell lines [58] and can also be used to generate cell cultures from PDX and organoid cultures. Finally, conditionally reprogrammed cells retain cell lineage commitment and maintain the heterogeneity of cells present in a biopsy [12,50,59,60,61,62]. In this article, we discuss application of CRC in patient-derived cancer models using tongue squamous cell carcinoma (TSCC) as an example.

## 4. An Example of the Matched Normal and TSCC Cancer Model using Conditional Cell Reprogramming (CR) Technology

Head and neck squamous cell carcinoma (HNSCC) ranks as the sixth most common cancer worldwide [63]. Historically HNSCC have been directly linked to long term tobacco smoking and alcohol consumption [64]. However, during the last decades this trend has changed, and the human papilloma virus (HPV), specifically the high risk HPV16, has been associated with an important percentage of the disease’s etiology giving rise to the HPV-related HNSCC [65]. Despite advancements in basic science and improvements in clinical treatment, the overall survival rate for patients with advanced HNSCC still remains less than 50% [66], possibly due to the lack of personalized cancer therapies that take into account the differences in tumor behavior from patient to patient as well as the patient’s response to treatment. Currently, the standard therapies for advanced head and neck cancer include surgery, radiation therapy and chemotherapy. Targeting therapies have not been benefit to patients with HNSCC since there are not well-established targets or therapies available. We will discuss the application of CRC technology in cancer research with a case with tongue squamous cell carcinoma (TSCC) as an example. 

### 4.1. Establishment of Patient-Derived Matched Normal and TSCC CRCs

The patient was 60-year-old white female, with pathologic T3N0 squamous cell carcinoma of the left tongue, non-smoker, non-drinker. Patient underwent a partial glossectomy. Tumor resected to positive margins at the deep aspect of the specimen. Tumor and adjacent normal tissues were dissected and digested with a mixture of F medium, collagenase, hyaluronidase and dispase. Then, digested again with 0.25% Trypsin-EDTA (Invitrogen, Carlsbad, CA, USA. Residual fat was removed by filtering the cell suspension through a 70 µm cell strainer (BD Biosciences, San Jose, CA, USA).

**Figure 1:**
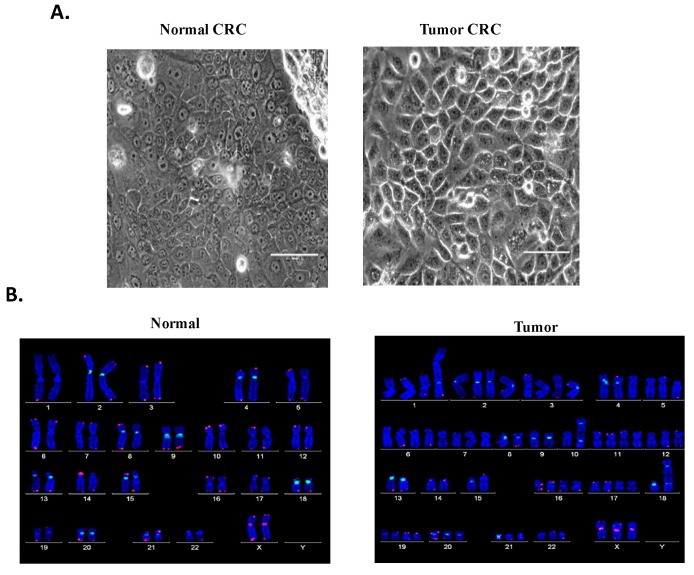
In vitro culture of matched normal and tumor conditionally reprogrammed cell cultures (CRCs). (**A**) Cell morphology: under light microscope the non-malignant cells appeared to grow in tight colonies (inner square) surrounded by feeder cells, a characteristic of cells cultured in CRC, whereas malignant cells grew as individual big cells (inner square) displaying prominent intercellular junctions. Non-malignant cells exhibited a heterogeneous population composed of small, dark, hexagonal cells and some big and flat, while tumor cells displayed a highly homogenous population of big, dark, hexagonal cells (outer squares). Bars: 50 and 200 µm. (**B**) Karyotype: cytogenetic analysis of normal and tumor CRCs were performed at early passage. The non-malignant line (46, XX) displayed a diploid karyotype with no apparent chromosomal alterations (left) whereas the malignant line consisted of a near-triploid clone karyotype (74, XXX, +1, +1, +1, +2, +2, +3, +3, +4, +5, +6, +6, +7, +7, +10, +10, +11, +11, +12, +16, +16, +17, +17, +18, +19, +19, +20, +21, +22) (right).

Normal and tumor CRC cells were cultivated and conditionally reprogrammed using the CRC approach [12,67]. Both cell lines were passaged for approximately 160 days and reached more than 80 population doublings during that period of time. At early passages, the non-malignant cells proliferated slightly faster than their malignant counterpart. However, in later passages both cell lines grew in a similar fashion. The normal CRC cells formed tight colonies surrounded by feeder cells (Figure 1A), similar to the previously established CRC lines [12,67] and exhibited a heterogeneous population composed of small, dark, hexagonal cells and some big and flat cells (Figure 1A). On the other hand, malignant cells displayed a unique phenotype consisting of bigger individual cells with prominent intercellular junctions (Figure 1A) and a highly homogenous population of dark, hexagonal cells. Karyotypic analysis of the CRCs performed at early passages resulted in a diploid karyotype (46, XX) with no apparent chromosomal alterations in the non-malignant line (Figure 1B). In contrast, the malignant line revealed a highly aneuploid karyotype with near-triploid clone and a modal number of chromosomes of 74 displaying aberrations in the majority of the chromosomes with variations that included number: trisomy 4, 5, 10, 12, 18, 20, 21, 22 and X; tetrasomy 2, 3, 6, 7, 11, 16, 17, 19 and pentasomy 1; as well as structural abnormalities such as fusions in chromosomes 3, 10, 18 (Figure 1B). 

### 4.2. Biological Characterization

#### 4.2.1. Organoid Cultures

**Figure 2:**
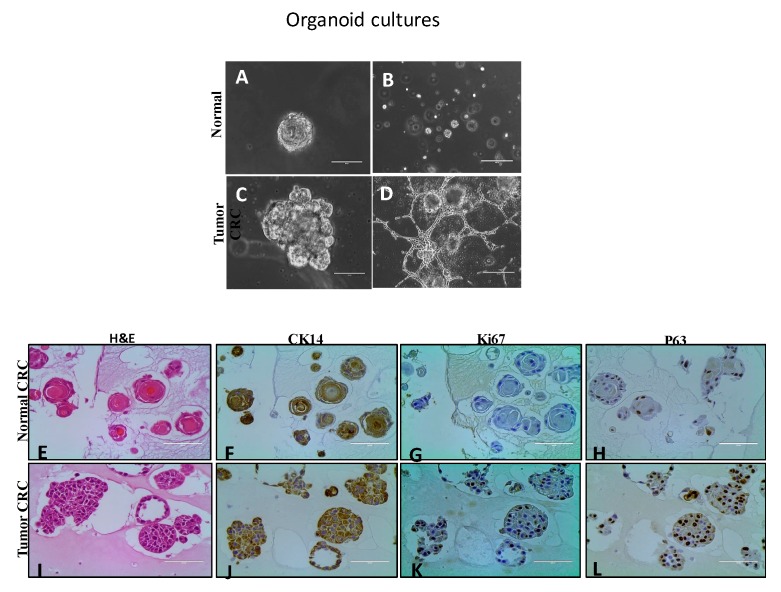
Organoid cultures of normal and tumor CRCs in matrigel. Morphogenesis: normal CRC (**A**) formed small spheres of approximately 100 µm characterized by displaying only one lobule with a mass-like shape and central growth, while tumor CRC (**C**) formed more prominent spheres of more than 200 µm consisting of multiple lobules with a grape-like shape and defined edges. Bars 100 µm. Unlike normal CRC that grew as independent spheres (**B**), tumor CRC developed invasive processes (**D**) which communicated adjacent spheres. Bars 400 µm. Immunohistochemistry: spheres from on-malignant cells depicted a polarized central growth with a structure similar to an arrested acinus (**E**) whereas the spheres from malignant cells were formed by actively proliferating cells (**I**) correlated with Ki-67 where the non-malignant displayed a negative staining (**G**) but stained moderately positive to P63 (**H**) while a high percentage of malignant cells stained strongly positive for Ki-67 (**K**) and the remaining for P63 (**L**). All the spheres from both lines stained strongly and diffusely positive for CK (cytokeratin) 14. Bars: 100 µm.

Historically, 3D cultures have represented an excellent system to discriminate malignant and non-malignant cells (30) and to recapitulate their structural organization. Using this model, the matched CRCs were plated using the embedded method and monitored for 10 days. Consistent with previous studies done on epithelial cells from different organs, mainly breast [68,69,70], here we found that both of our matched CRCs successfully formed spheres that shared similar phenotypes as those formerly reported. The non-malignant cells gave rise to small spheres of approximately 100 µm characterized by displaying only one lobule with a mass-like shape and polarized growth (Figure 2); while their malignant counterpart was distinguished for forming prominent spheres of more than 200 µm consisting on multiple lobules with a grape-like shape and defined edges. Unlike normal CRCs that grew as independent spheres, at day 8 the CR tumor cells formed spheres started developing invasive processes which communicated adjacent spheres which by day 10 covered approximately 50% of the well surface. With less than 4% of the publicly available HNSCC lines being able to congregate into spheroid clusters [71] and with no reports of any normal line being able to do so, this result constitutes the first report of this kind where both HN donor matched pair cell lines were able to proliferate and efficiently re-associate into spheroids in a 3D system.

Hematoxylin and eosin staining (H&E) revealed that the great majority of spheres from the non-malignant line consisted in a polarized central growth and formed structures similar to arrested acinus with a well-defined lumen (Figure 2). Instead, the spheres from the malignant line consisted of actively proliferative cells which failed to arrest growth and lost polarity (Figure 2). Likewise, immunohistochemistry of specific molecular markers of the tumor CRCs cultures showed that the normal CRC cells stained negatively to the proliferation marker Ki-67 (33) (Figure 2) and moderately positive P63 (Figure 2) a well-recognized differentiation marker [72], while the malignant cells stained moderately positive to Ki-67 (Figure 4) as well as P63 (Figure 2). All the spheres developed by both cell lines stained strongly and diffusely positive for CK (cytokeratin) 14 (35) (Figure 2), confirming the squamous epithelial origin of the cells.

#### 4.2.2. Transformation and Tumorigenicity Assays

**Figure 3:**
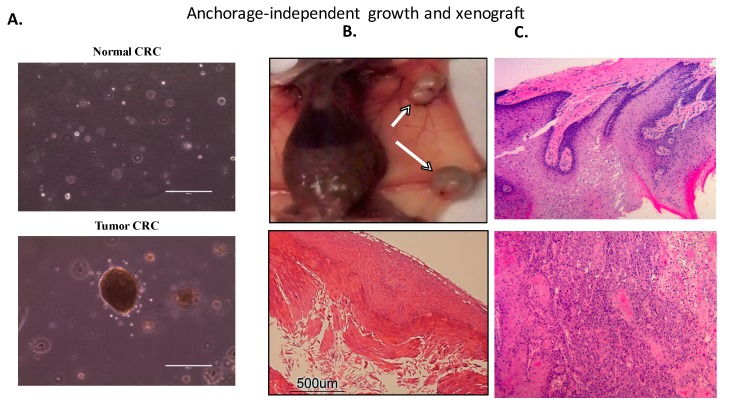
**Tumorigenicity** Assays. (**A**) Soft agar colony formation assay: in an anchorage-independent growth assay only tumor CR cells formed visible spheres after two weeks in soft agar culture. Scale bars: 500 µm. (**B**) Xenografts: tumorigenic properties of tumor CR cells were defined by an in vivo assay. Six-week-old athymic mice were inoculated with 1 × 10^6^ normal or tumor conditional cell reprogramming (CR) cells (left). The resulting tumors were resected after 3–3.5 months of injection and stained with Hematoxylin and eosin (H&E) (middle) staining (right). (**C**) Tumor growth: tumor CR cells formed palpable tumors three weeks after injection in both flank and mammary sites displaying similar growth rates and patterns.

In order to evaluate the ability of the matched CRCs to originate growing tumors, we set up an in vivo assay using an adapted protocol by means of athymic nude mice, the most widely used mouse model for the HN cancer studies [73]. Normal and tumor CRC cells were heterotransplanted to mice, forming palpable xenografts only in sites injected with malignant cells with a 100% tumor formation rate (10/10 sites) (Figure 3). No palpable xenografts were produced in sites injected with cells cultured from non-malignant tissue (0/10 sites). Palpable tumors first appeared three weeks after injection in both mammary gland (*n* = 6) and flank sites (*n* = 4) (Figure 3). Importantly both xenografted sites showed very similar tumor growth curves, evidencing that the tumor development was not site specific and that the cells did not show any type of tissue tropism; instead, the cells themselves possessed a high tumorigenic potential. All mice were necropsied ~3 months following flank injections when the palpable xenografts reached between 1 and 1.4 cm^3^ in size as the mice that received mammary injections were necropsied 3–3.5 months when the palpable xenografts reached between 1 and 1.6 cm^3^. Even though some authors argue about the feasibility of xenografts produced by cells grown in vitro, since they may had adapted different phenotypes while cultured in plastic [71], here we found that the histologic sections of the xenografts originated from our matched CRCs displayed well differentiated squamous carcinoma (Figure 3) faithfully resembling the morphology and histologic characteristics of the original tissue (Figure 3).

In addition, we also evaluated the ability of our matched CRCs to grow in soft agar since cell proliferation in this system has been strongly associated with in vivo tumorigenic growth potential. Consistent with our xenograft experiments, after two weeks in soft agar culture, only the malignant line was able to proliferate and develop colonies (Figure 3) in an anchorage-independent manner confirming its transformation as wells as its uncontrolled growth, fundamental properties of the malignant cells [74].

### 4.3. In Vitro Chemosensitivity of Matched CRCs

**Figure 4:**
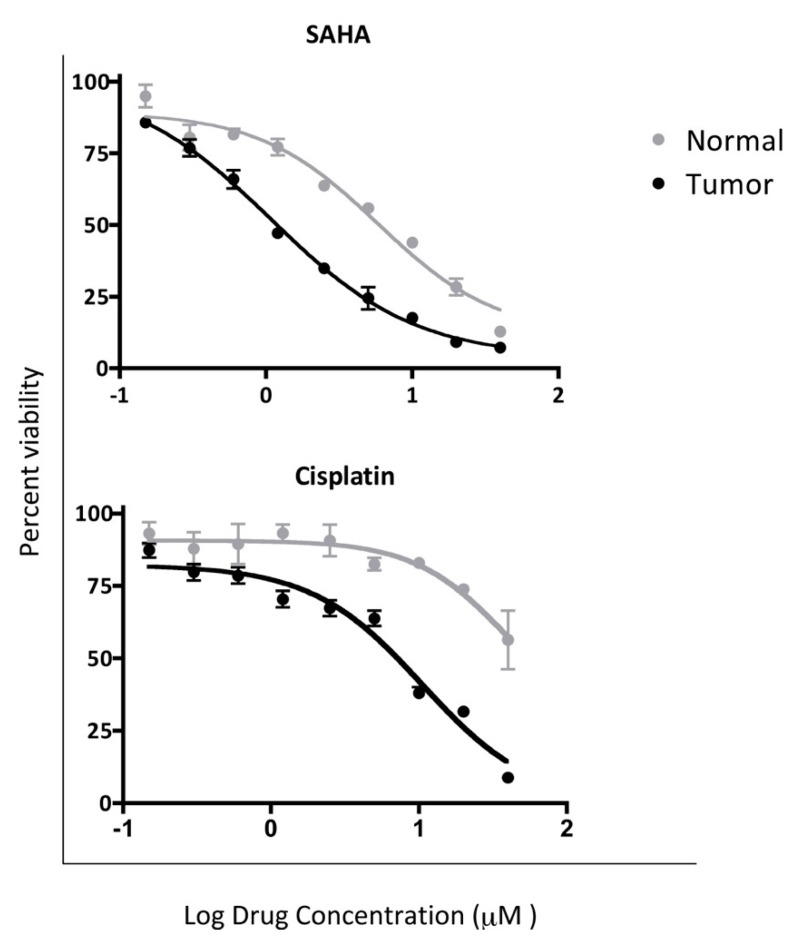
In vitro chemosensitivity of normal and tumor CRCs. Differential toxicity of SAHA and cisplatin was established in the matched normal and tumor CR cells via ATP bioluminescence. Malignant cells showed more sensitivity to both compounds by displaying a median curative dose of 1.09 µM for Vorinostat (SAHA) and 10.47 µM for cisplatin compared to 5.83 µM and 39.91 µM respectively for the normal line (*p* values: 0.011 and 0.014).

In order to determine the differential toxicity of the normal and tumor CRC cells to single chemo agent approved for the treatment of HNSCC, we measured the cell viability after 48 h of treatment in concentrations ranging between 0–40 µM of Vorinostat (or SAHA: suberoyl+anilide+hydroxamic acid), Cisplatin via ATP bioluminescence using an adapted protocol [75]. From the tested compounds, only Vorinostat and cisplatin successfully inhibited cell proliferation of the malignant cells and had limited effect in the non-malignant line (Figure 4). Tumor CRCs were more sensitive to both compounds showing median curative dose of 1.09 µM for Vorinostat and 10.47 µM for cisplatin respectively compared 5.83 µM and 39.91 µM for normal CRCs.

### 4.4. Top Active Pathways in the Tumor CRC Cells

**Figure 5:**
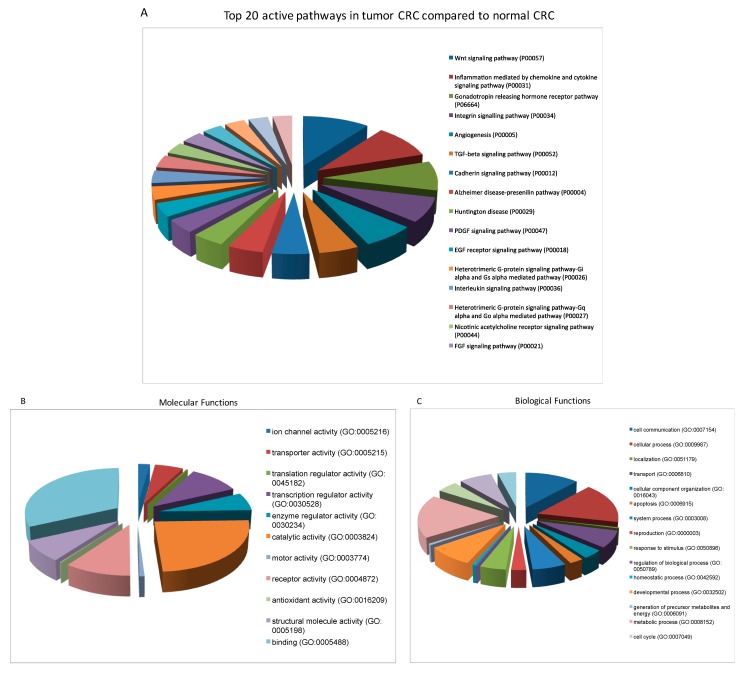
Top 20 active pathways (**A**) and their distributions of molecular (**B**) and biological (**C**) functions in tumor CRC. RNA sequencing analysis showed that the angiogenesis, Wnt, integrin, inflammation mediated by chemokine and cytokine as well as the gonadotropin releasing hormone receptor pathways were the most highly active in tumor CRCs.

Aiming to uncover the possible gene expression variance between the normal and tumor CRC cells, which could be responsible for their phenotype, behavior and the overall development of the disease, we set up an RNA-seq experiment and performed a pathway analysis. A number of pathways differed between the two cell lines and were particularly active in the malignant line. However, in this report we only focused in the five of them, the one that showed the greatest variance (Figure 5). Not surprisingly, three of these pathways have been extensively reported to be altered in high percentages in different types of cancers. Overactivation of those signals has been associated with cancer promotion and development through neovascularization and ultimately enhancement of tumor survival and progression (angiogenesis pathway) [76]; driving the self-renewal and tumorigenicity of tumor stem-like cells (Wnt pathway) [77] and promoting malignant cell proliferation and decreasing their differentiation (Integrin signaling) [78]. On the other hand, the remaining two pathways are not commonly altered in cancer but have been encountered in some malignancies including HNSCC. This way, the gonadotropin releasing hormone receptor pathway has been shown to be involved in different aspects of cell behavior in extra-pituitary tissues like HN and its alteration has been related to tumor cell proliferation, increase of motility and metastasis [79]. Meanwhile, alterations of the inflammatory pathways have been limited to chronic infections and inflammation; and in this particularly case, its overactivation could be due to chronic inflammation caused by smoking factor that not only plays a role as a tumor initiator but as a promoter as well [80].

The development of patient-derived matched CRCs is an invaluable tool for cancer research and for the development of targeted therapies, especially if these lines preserve the biological, molecular, genetic and histological characteristics of the original tissues. Our data demonstrates that our CRCs-based biological model allows us to recapitulate TSCC, constituting the most accurate method to investigate in a comprehensive manner the condition and perform in vitro tests that could be later translated into the clinical setting without the adverse effects of the in vivo tests.

## 5. Next-Generation Living Biobanks (NGLB)

Biobanks provide a platform for innovative biomedical research. Recent scientific advances in cryopreservation have enabled the prospect of establishing “living biobanks” that store viable, functional tissue or replicable cell types for years to decades [37,81,82,83,84]. This will have a significant impact across basic biological research, medicine and the biopharma industry; however, the effects of such applications are underexplored. Recent approaches, for example, patient-derived xenograft models, organoids, conditionally reprogrammed cells, induced pluripotent cells, and other cancer precision medicine applications, represent an unexhausted resource of living biobanks. However, these concepts are applied in very many different ways by the academic and private sectors, representing an actively growing field that has yet to reach clinical consensus or maturity. Here we summarize application of CR technology in living biobanks.

**Figure 6:**
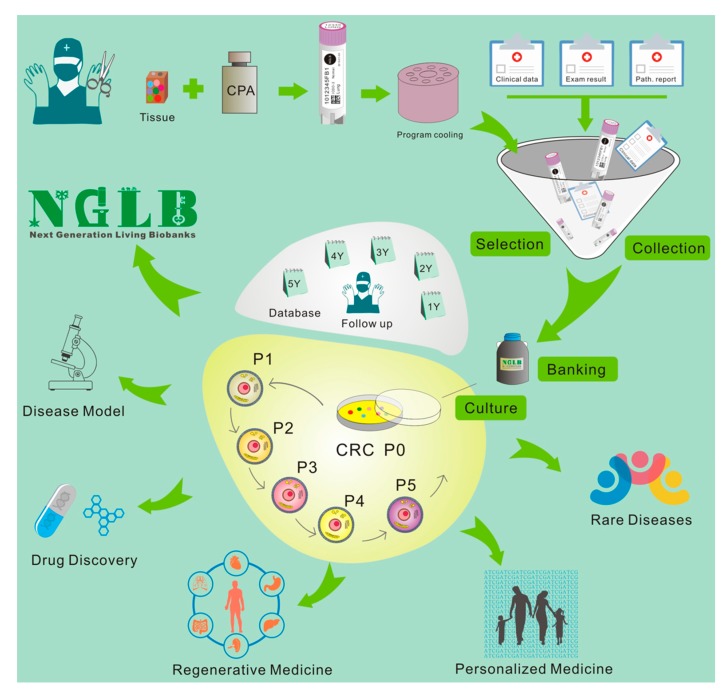
Workflow of next generation living biobanks (NGLB). Specimens including surgical specimens, core biopsies, needle biopsies, brushed cells, or cells from liquid biopsies (blood, urine, etc.) from patients with disease and/or different stages of disease (for example, before and after treatment, primary or metastatic, etc.) will be collected, cryopreserved, and stored together with their corresponding -omics (genomic, transcriptomic, proteomic, metabolomic, etc.) and clinical information. CR technology will be used to generate an unexhausted cell cultures for living cell banks for future demands in drug discovery, disease modeling, regenerative medicine. CR cells can be used to generate other types of living biobanks such organoids or patient-derived xenografts (PDX) (for tumor).

28–29 March 2011, we first presented “bring biobank to life” at BRN 2011 Symposium “Advancing Cancer Research Through Biospecimen Science” of NCI when we started CR technology [12,84]. Editor in Chief of American Journal of Pathology proposed applications of CR technology in living biobanks [84]. Eventually, more and more articles came out for tumor living biobanks as the applications of CRC and organoid cultures [37,43,46,51,59,85,86,87,88,89,90,91,92,93,94,95,96,97,98,99,100,101,102]. However, that will be impossible task in terms of efforts, work load and cost if one would like to establish living biobanks from every patient in the hospital for the immediate use of personalized medicine. In order to balance of distribution of disease types for research and industry, it’s extremely important to cryopreserved tissue specimens and will generate cell cultures using CR and/or organoids technologies. Thus, we brought forth a new concept: next generation living biobank (NGLB) (Figure 6). Except for immediate use of personalized diagnostics and treatment, we suggest to collect and cryopreserve specimens including surgical specimens, core biopsies, needle biopsies, brushed cells, or cells from liquid biopsies (blood, urine, etc.). These specimens should be collected and stored together with their corresponding -omics (genomic, transcriptomic, proteomic, metabolomic, etc.) and clinical information. Then, CR technology will be used to generate an unexhausted cell cultures for living cell banks for future demands in drug discovery, disease modeling, regenerative medicine. These cryopreserved specimens, their corresponding CR cells and CRC- derivatives together compose of NGLB (Figure 6). Compared to regular living biobanks, this NGLB strategy would be a highly efficient platform with the minimal time, money, and other support, this may also avoid redundant cell lines from common diseases, for example, lung cancers. Most importantly, this NBLG includes cryopreserved specimens with broader coverage of disease types, which allow us to generate cell cultures from rare disease using CR or organoids cultures, since there are no available cell models or very few cell lines for many rare diseases, for example, adenoid cystic carcinoma (ACC), neuroendocrine cancer, etc. We also list properties of conventional cell lines, CRC, organoids and PDX in basic and translational cancer research (Table 1).

## 6. Applications of CR-based NGLB

### 6.1. Precision Medicine

We discussed above that omics-based precision medicine help very limited number of the patients with cancer. When we established CR technology, we initiated a real case study on recurrent respiratory papillomatosis (RRP). A patient with RRP with chemo-resistant and progressive disease [103]. We were able to obtain both tumor and normal tissues from the patient and generate paired CR normal and tumor cell lines for drug screening. As a result, vorinostat showed a significant cytotoxic effect on CR tumor cells compared to the normal cells. Importantly, the patient received a three-month course of treatment with vorinostat and demonstrated a stable disease [103]. Therefore, CR greatly facilitated a rapid expansion of primary cells without changing their genetic profile and in vitro drug testing for individual patient. Currently, CR cultures have been used for drug testing for patients with lung cancer, salivary gland cancer, bladder cancer, and breast cancer. For example, CR lung cancer cells from 14 cases were successfully generated and characterized, with six cases tested with nedaplatin, cisplatin, carboplatin and vinorelbine demonstrating consistency with clinical scenario [104]. Similarly, CR cells from primary tumors enabled rapid screening of candidate drugs and facilitated individualized treatment [59,96,101,105,106]. CR cells and mouse xenograft model can be used for the matched in vitro and in vivo drug testing for the patients [107]. Most recently, Kettunen K et al. reported the generation of patient-derived CR bladder cancer (BC) cells for personalized drug testing [91,108,109,110]. Since BC patients suffer cancer recurrence of over 60% at two years and progression into advanced stages in up to 25% of patients, almost all patients need long-term intrusive, uncomfortable, expensive cystoscopy which makes bladder cancer the costliest cancer (per case) of all cancer types. Due to the requirement for invasive biopsies to generate these tumor CR cells, there are limited clinical applications. To overcome this limitation, Jiang S, et al. successfully established CR cells from urine samples of BC patients with a success rate of 80–90% [92]. This non-invasive approach allows for monitoring cancer recurrence and for predicting efficacy of treatment of BC: 1) to predict or detect recurrent bladder tumors earlier; 2) to monitor responses to immunotherapies; 3) to predict responses to neoadjuvant chemo/targeted therapies; and 4) to identify new drugs for first line or second line BC treatment. CR technology has the potential to transform the clinical management of BC patients [91,109,110,111].

CR technology can be used for novel therapeutic strategies such as combination of drugs. For example, CR cells identified LA-12 enhanced cell death by TRAIL [112], and combinational treatment of TRAIL with cisplatin/LA-12 killed prostate cancer cells more effectively [113]. Patient-derived CR models of tyrosine kinase inhibitors-acquired resistance of NSCLC enabled the screen of novel active drug combinations [114]. Thus, CR technology can serve as a novel functional diagnostic platform for precision medicine.

### 6.2. Regenerative Medicine

Directed differentiation of embryonic or induced stem cells with high efficiency to functional cell types bas been very difficult [115]. Since normal CR cells can differentiate well when CR conditions removed and CR cells were transferred to in vitro or in vivo differentiation conditions [12,50,51], suggesting potential of CR cells in tissue repair or regenerative medicine. CR esophageal epithelium with same pattern of gene expression and phenotype can be implanted into the esophagus for repair or replacement of the damaged region for the treating eosinophilic esophagitis [116]. Combination of CR airway cells with lung fibroblasts facilitated establishment of a 3D tracheosphere culture scaffold [117], this was biocompatible when engrafted into decellularized trachea of the rabbit model [117]. The CR airway cells were also differentiated into both upper airway bronchial epithelium and lower airway alveolar structures after implantation into the decellularized mouse lung [118]. These studies indicated the potential of CR airway cells in airway reconstruction and lung transplantation [119,120,121]. Importantly, the fresh bronchial biopsy samples can be frozen for transport, which facilitates to minimize the inconvenience by the distance from a hospital for sampling bronchial biopsy and the laboratory capable of cell culture. Several researchers applied the CRISPR-Cas9 gene editing technology on CR cells to investigate molecular mechanisms. For instance, CRISPR-Cas9 genetic editing of CR cells revealed the pro-inflammatory effects of MUC18 in airway epithelial cells and the role of the NLRP1 Inflammasome in UVB sensing in human primary keratinocytes [122,123]. The genome editing may promote more studies and potential medical applications in using CR technology.

Therefore, CR based rapid expansion of patient-derived cells with stable genotypes will meet an unmet need in tissue engineering for individualized regenerative medicine.

### 6.3. Modeling Diseases

CRC cells can be established from both pathological and pathological specimens, these CRC cells can be further cultured in 2D and 3D culture systems. Most studies on CR have been on variant of cancer types. As we described above, many cancer studies use stable cell lines [124,125,126,127], which cannot represent both inter-tumor and intratumor heterogeneity of tumor cells within tumor microenvironment [128,129,130]. Tumor CR cells can be directly generated in 2D culture or spheroids or organoids [51,98]. Importantly, CR tumor cells formed CR-cell derived xenografted (CDX) tumor when they were implanted to mice. Brown et al. established patient-derived CR cells from 19 ductal carcinoma in situ (DCIS) tumors. These CR cells maintained both luminal and basal cellular features with tumor heterogeneity, and reflected progression of DCIS [62]. CR cells from non-small cell lung cancer (NSCLC) tumors also demonstrated tumor heterogeneity in cultures, suggesting a preclinical model for lung cancer studies [131]. ACC is a rare salivary gland cancer with high metastatic potential, currently there is no reliable model for this rare cancer [132], Chen et al. successfully generated a coupled in vitro and in vivo models (mouse and zebrafish), CR cells and CR zebrafish models, from ACC PDX tumors [101]. CR cells from PDX tumors with human lung, ovarian or bladder tumors maintained the original genetic alteration and response as their parental mouse models [88,102]. CR cell lines from the C57BL/6 MYC driven prostate adenocarcinoma expressed markers of luminal epithelial lineage and provided a model for MYC-driven prostate cancer [133]. Given the long latency of tumor formation in the animal models, CR cells also were used for establishing tumor cell lines from both UPPL and BBN tumors [94].

Airway epithelial cells play an essential role in maintaining airway immune responses, while these epithelial cells in newborns/infants are difficult to obtain [134,135]. Brewington et al. established CR nasal epithelial cells and measured activity of CFTR for pulmonary edema study [136]. A similar study also used in pig model [126]. CR cell models were also used for the nasal airway epithelial tissues of infants and young children in vitro [137]. These CR cells demonstrated appropriate immune response as well as induction of inflammatory response, can be used to immune-biology and pathogenesis of respiratory disorders in children [137]. CR mouse intestinal epithelial cells from both wide type and *Apc*^Min/+^ mouse retained large numbers of genotype-specific cell models [138]. CR cells have been used for establishing tumor cell lines from mouse bladder and kidney cancer models [94,139,140]. Most recently, Su et al. established both diseased and normal human primary hepatocytes using CR technology [141,142]. All these studies demonstrated the wide applications of CR technology in disease modeling.

### 6.4. Discovery of Novel Targets and Drugs

CR Technology can serve a novel platform for drug discovery in two aspects. As we discussed in diseases modeling section, CR can be used to evaluate efficacy of new drugs. Since CR normal cells can be differentiate properly in 3D conditions and in vivo environments. This suggests that CR normal cells can be also used for evaluation of drug toxicity. For example, CR cells were established from PDX models with pancreatic ductal adenocarcinoma cells [60], a novel potential therapeutic target, ERCC3-Myc interaction, and a covalent inhibitor of ERCC3, triptolide, have been identified from CR cells [60]. CR cells of ACC expressed high levels of Notch1 and Sox10, and were highly tumorigenic in mice [143]. DAPT, a γ-secretase inhibitor, selectively inhibited growth of CR ACC cells in vitro and in vivo through inhibition of Notch1 [143]. Pollock et al. evaluated the anticancer effect of the natural product strigolactone analogues using matched CR prostate normal and tumor cells, showing that strigolactones selectively killed prostate cancer cells [144]. Kim et al. identified that an anticancer candidate IDF-11774 interacted with ATP6V0C and synergized with the ATP6V0C inhibitor, bafilomycin A1, to inhibit CR colorectal cancer cells with low Bcl-2 expression [145], potentiating IDF-11774 for further clinical trials. The CR vaginal epithelial cells served as an in vitro model for assessment of drug effect [146]. Several groups have used CR cells for high through put drug screening [59,91,92,147].

Since CR technology can be used for propagation of both primary normal and pathological cells, the CR cells as a model can be applied for assessment of either drug efficacy or toxicity. CR cells of normal limbal epithelial cells were used to evaluated for drug toxicity assessment [54]. CYPs are important drug metabolizing enzymes in human, and neither transiently cultured normal cells nor immortalized cell lines cannot maintain high expression level of CYPs. CR human normal bronchial epithelial cells expressed considerable level of cytochrome P450 enzymes (CYPs), and benzo(a)pyrene significantly induced CYPs expression in CR cells [148]. Su et al. also showed that CR hepatocytes expressed CYPs [142]. Therefore, CR can provide a valuable in vitro model for toxicity evaluation and drug metabolism.

### 6.5. Others

CR has been largely used to generate human stable cell lines [12,50,51,88,90,92,95,96,97,98,99,100,114,130,141,149,150,151,152,153,154,155,156,157,158,159,160,161,162,163,164,165] and several researchers have applied CR for establishment of stable cell lines from mouse [52] fish [53], pig [54], horse [55,56], rabbit [54], and dog [57]. For instance, Gardell et al. reported that immortalized cell lines from brain and lip epithelia of Mozambique tilapia (Oreochromis mossambicus) were established using CR method [53].

## Figures and Tables

**Table 1:** Comparison of a patient-derived cancer models.

	Conventional Cell Lines	Organoids	PDX	CRC
Sample size				
FNA	−	+/−	−	+++
Core Biopsy	−	+	−	+++
Surgical Specimens	+	+++	++	+++
Timing	dozen days	1-5 weeks	1-5mont	1-10 days
Success rate of initiation	+ 0–10%	++ (5–80%)	++ (2–30%)	+++50–100%
Tumor type specific				
Rapid Expansion	+++	++	+	+++
Matched Normal con	−	+	-	+
Karyotypic stability	−	++	N/A	++
3D growth	−	+	+++	−
Representation of tumor	+	++	++	++
Genetic manipulation	+++	++	−	++
Maintenance (passage)	+++	++	+	+++
LT drug screens	+++	–	+	+++
HT drug screens	+++	++	−	+++
Heterogeneity	−	++	+++	++
Tumor–stroma interaction	−	−	++	−
